# Novel insights into the role of eye movements during REM sleep in memory consolidation

**DOI:** 10.1093/sleep/zsad178

**Published:** 2023-07-11

**Authors:** Michele Ferrara, Aurora D’Atri, Federico Salfi

**Affiliations:** Department of Biotechnological and Applied Clinical Sciences, University of L’Aquila, L’Aquila, Italy; Department of Biotechnological and Applied Clinical Sciences, University of L’Aquila, L’Aquila, Italy; Department of Biotechnological and Applied Clinical Sciences, University of L’Aquila, L’Aquila, Italy

A large and consistent literature converges in pointing out how hippocampus-dependent memories benefit from slow wave activity during non-rapid eye movement sleep (NREM). The declarative memory traces are repeatedly reactivated during slow-wave sleep (SWS), promoting long-term storage at the cortical level [1, 2]. Sleep also supports the consolidation of procedural memory underlying problem-solving and the acquisition of new rules or cognitive strategies [3–5], but in this context, the specific role of SWS is more controversial. While some studies support the possible involvement of SWS [6, 7], others propose rapid eye movement (REM) sleep as the main responsible for the sleep-dependent integration of information supporting creative problem solving [8]. Meanwhile, the iterative and synergic interleaving of REM and NREM stage characteristics has been recently conceptualized to explain the sleep effect on creative problem-solving skills [3]. However, evidence of specific cortical processes representing the actual reactivation of this kind of memories during paradoxical sleep is still scarce.

Another gap in the literature addressing the relationship between REM sleep and memory function is represented by the common practice of considering paradoxical sleep as a homogeneous state, especially in human studies [9]. However, from decades, two neurophysiologically distinct REM substates have been identified [9, 10], namely the tonic and phasic REM sleep, and the available literature suggested a functional heterogeneity of these two substates in memory consolidation [11–13].

In the August issue of *SLEEP*, van den Berg and collaborators [14] addressed some of these open and scarcely explored questions, providing novel insight into the role of the electroencephalographic (EEG) activity during human tonic and phasic REM sleep in the consolidation of novel problem-solving skills. Two different groups of healthy young participants (*n* = 20 per group) performed the Tower of Hanoi task before and after (1) an undisturbed 8-hour period of sleep, or (2) a same-length wake period. Each group also took part in another similar condition (in counterbalanced order) in which a non-learning control task was performed. The study [14] adopted a quite novel approach to address the involvement of REM sleep in memory consolidation, evaluating the event-related spectral perturbations (ERSP) time-locked to the eye movement (EM) peaks. This analysis allowed the authors to estimate specific oscillatory EEG components surrounding EMs during REM sleep, unraveling stable cortical patterns associated with EMs in the post-learning night compared with the control (non-learning) condition. Furthermore, the authors [14] classified the EMs during REM sleep into “bursts,” defining phasic REM state, and “isolated” EMs, proposed as a proxy of tonic REM state. This categorization led to evaluating the differential contribution of phasic and tonic REM sleep to the consolidation of problem-solving skills.

Besides confirming the beneficial effect of sleep on problem-solving performance [3, 4], the study highlighted an intriguing relationship between sleep-dependent behavioral improvement and the EEG correlates of EMs that characterize post-learning REM sleep. In detail, the patterns accompanying EMs during post-learning phasic REM sleep, as compared to a control night, included increased oscillations in the 2–16 Hz range following the EMs peak, which differed as a function of scalp location and frequency (prefrontal ~2 Hz, central ~7 Hz, occipital ~8 Hz, and ~16 Hz). During post-learning tonic REM sleep, EMs were preceded by increased activity in the 9–14 Hz range in central (~9 Hz, ~14 Hz), parietal (~13 Hz), and occipital areas (~4Hz, ~6 Hz, and ~12 Hz), and followed by increases at ~3 and ~6 Hz in prefrontal and occipital regions, respectively. Interestingly, the changes in the oscillatory activities of phasic REM sleep were correlated with the overnight changes in behavioral performance (speed/accuracy), while no significant association has been found for tonic REM sleep.

Overall, the presence of post-learning variations in the EEG activity time-locked to EMs during REM sleep indicates an engagement of the cortical processes that occur during EMs in the procedural memory consolidation. This evidence involves both phasic and tonic REM periods, albeit only those changes that occurred during phasic REM periods were predictive of the improvement in cognitive performance, suggesting a different functional role of the two substates in the sleep-dependent consolidation processes.

In discussing the results, the authors [14] interpreted the cortical activity in the ~8–16 Hz range as *sensorimotor* rhythms (SMR), although others have defined the same activity as *Mu* [15]. Moreover, all the activity in the ~2–8 Hz range has been defined as *theta* rhythm, thus including low frequencies (2–4 Hz) belonging to the delta band. Following this choice, the discussion of the findings involving the < 8 Hz activity has been mainly focused on the available evidence linking REM theta oscillations to memory consolidation, although in most human studies a standard 5–8 Hz definition of the theta band has been adopted. From a different perspective, it could be interesting to provide an alternative interpretation of the post-learning increase in the 2–4 Hz activity found in the prefrontal region. We might look at these results in light of the possible implication of the 2–4 Hz EEG rhythms during phasic REM sleep in memory consolidation. Recent studies indeed demonstrated the existence of slow oscillations in this frequency range during paradoxical human sleep, and defined them as *sawtooth delta* waves [16, 17]. These EEG rhythms have been proposed as the human analog of the ponto-geniculo-occipital waves described in animals, and investigations combining stereo-EEG and polysomnography suggested an active role of these waves in orchestrating synchronized reactivations and the coordination with deep brain structures functionally involved in memory consolidation [16]. In this view, the neural correlates of the reactivation during REM sleep would involve three different brain rhythms: prefrontal sawtooth delta waves, an occipital theta rhythm, and a centro-parieto-occipital SMR, enriching the scenario proposed by the authors.

In conclusion, this study provides tantalizing clues about the role of paradoxical sleep EMs and their concomitant neocortical activations in the consolidation of problem-solving skills.

The application of the ERSP approach to sleep EMs paves the way for future investigation to explore the involvement of EMs and their correlates in several sleep-dependent cognitive domains, disentangling the differential contributions of tonic versus phasic REM states not only in memory consolidation, but also in dreaming or REM-related sleep disorders.

The present study raises as many questions as it answers, highlighting the need for future research. What drives the differential involvement of delta, theta, and SMR activity during phasic and tonic REM sleep? Can these findings be generalized to other types of procedural learning? What are the implications for sleep disorders or conditions characterized by REM sleep alteration, such as in aging [18], post-traumatic stress disorder [19], neurodegenerative diseases [20], or partial sleep deprivation? Furthermore, in the light of a possible complementary engagement of REM and NREM sleep characteristics [3], future research should evaluate whether and how the brain oscillation correlates of EMs may interact with the hallmark NREM rhythms involved in memory consolidation processes, such as slow oscillations and spindles [1, 2]. We look forward to watching the field grow, one eye movement at a time.
